# Unveiling the cell dynamics during the final shape formation of the tarsus in *Drosophila* adult leg by live imaging

**DOI:** 10.1007/s00427-024-00719-z

**Published:** 2024-07-08

**Authors:** Shotaro Hiraiwa, Shumpei Takeshita, Tensho Terano, Ryuhei Hayashi, Koyo Suzuki, Reiko Tajiri, Tetsuya Kojima

**Affiliations:** 1 Department of Integrated Biosciences, Graduate School of Frontier Sciences, The University of Tokyo, Biosciences Building 501, 5-1-5 Kashiwanoha, Kashiwa-shi, Chiba, 277-8562 Japan; 2 Present address: Laboratory for Extracellular Morphogenesis, Graduate School of Science, Chiba University, 1-33 Yayoi-cho, Inage-ku, Chiba-shi, Chiba, 263-8522 Japan

**Keywords:** *Drosophila melanogaster*, Tarsus, Live imaging, Cell dynamics, Hemocyte, Basement membrane

## Abstract

Organisms display a remarkable diversity in their shapes. Although substantial progress has been made in unraveling the mechanisms that govern cell fate determination during development, the mechanisms by which fate-determined cells give rise to the final shapes of organisms remain largely unknown. This study describes in detail the process of the final shape formation of the tarsus, which is near the distal tip of the adult leg, during the pupal stage in *Drosophila melanogaster*. Days-long live imaging revealed unexpectedly complicated cellular dynamics. The epithelial cells transiently form the intriguing structure, which we named the Parthenon-like structure. The basal surface of the epithelial cells and localization of the basement membrane protein initially show a mesh-like structure and rapidly shrink into the membranous structure during the formation and disappearance of the Parthenon-like structure. Furthermore, macrophage-like cells are observed moving around actively in the Parthenon-like structure and engulfing epithelial cells. The findings in this research are expected to significantly contribute to our understanding of the mechanisms involved in shaping the final structure of the adult tarsus.

**Supplementary Information:**

The online version contains supplementary material available at 10.1007/s00427-024-00719-z.

## Introduction

Organisms exhibit a tremendous variety in their shapes. Understanding the mechanisms forming the various organismal shapes has been one of the important subjects in biology. Over the past several decades, considerable progress has been made in elucidating the mechanisms that determine cell fate during development. This has led us to understand that the basic mechanisms of cell fate determination are conserved among closely related species. In contrast to the mechanisms of fate determination, how fate-determined cells form final shapes remains elusive. Since shapes can differ dramatically between closely related species that are expected to share the basic mechanisms of cell fate determination, differences in final shape formation processes should greatly contribute to making the shape differences. Thus, understanding the mechanisms of final shape formation is of great importance to understand the mechanisms of formation and diversification of organismal shapes.

Insects account for more than half of living species and show extreme diversity in their shapes (Snodgrass 1935; Grimaldi and Engel 2005). This makes insects attract the attention of many researchers. Insect legs have also evolved into various shapes to adapt to various environments. Generally, they consist of several segments, which are the coxa, trochanter, femur, tibia, tarsus, and pretarsus, in a proximal to distal direction. Among these segments, the tarsus, located near the tip of the leg, is unique in that it is further subdivided into several segments called tarsal segments or tarsomeres. The number of tarsal segments varies between species and ranges from one to five. Even within the same individual, it sometimes varies depending on the developmental stage and on the body segments in which the leg is formed (Angelini et al. 2012). In addition to the number of tarsal segments, the shapes of tarsal segments are also variable. For example, the tarsus of the first leg in the male diving beetle is flat and expanded, making a fan-shaped sucking disc, while that in mosquitos is very long and slender (Kojima 2017). This morphological diversity makes the insect tarsus a good model for studying the mechanisms of final shape formation and its diversification.

In the adult leg of *Drosophila melanogaster*, the tarsus consists of five segments, the tarsal segments 1–5, from proximal to distal direction. The adult leg develops from the leg disc, which is a mono-layered epithelial sheet formed during embryogenesis. During the larval stage, the leg disc becomes a sack-like structure, part of which forms the disc proper that differentiates into the adult leg, while the other part becomes the peripodial membrane that covers the disc proper. With the growth of the leg disc, the disc proper is subdivided into concentric regions corresponding to the adult leg segments through the exquisite regulatory interaction of patterning genes. (reviewed in Kojima 2004, 2017; Estella et al. 2012; Ruiz-Losada et al. 2018) Accordingly, the most central part corresponds to the most distal segment (pretarsus), and the most peripheral part corresponds to the most proximal segment (coxa). Cells that develop into each tarsal segment are determined by the end of the late third instar. During these processes, the leg disc is folded and remains covered by the peripodial membrane. After the third instar, the larval cuticle changes to form the puparium, within which pupal development proceeds for about 4 days. Immediately after the puparium is formed, imaginal discs begin to join together and form the adult body. By 12 h after puparium formation (APF), the pupal cuticle is formed and encloses the developing adult tissue. During these processes, the leg disc everts and elongates with the breakage and retraction of the peripodial membrane (Proag et al. 2019).

Around the puparium formation, each tarsal segment is already recognizable morphologically by the epithelial folding or constriction between tarsal segments. The development of the tarsus by this stage has been extensively studied (reviewed in Kojima 2004, 2017; Ruiz-Losada et al. 2018). Regions corresponding to individual tarsal segments are determined by the region-specific expression of genes encoding transcription factors and their regulatory interactions (Kojima et al. 2000; Kozu et al. 2006; Tajiri et al. 2007; Pueyo and Couso 2008; Greenberg and Hatini 2009; Natori et al. 2012). This leads to the activation of Notch signaling in the distal end of each tarsal segment through regulation of the expression of Notch ligands, Delta, and Serrate, just proximal to the Notch active region in each tarsal segment (de Celis et al. 1998; Bishop et al. 1999; Rauskolb and Irvine 1999; Rauskolb 2001; Córdoba et al. 2016). This Notch activation induces the region-specific regulation of the cytoskeleton and cell death, resulting in the formation of constrictions between tarsal segments (Córdoba and Estella 2014, 2018; de Madrid et al. 2015; Monier et al. 2015). Recently, the involvement of the tissue-wide mechanical force generated by cell proliferation has also been suggested to contribute to the formation of constrictions in concert with the Notch signaling pathway (Rodríguez et al. 2024).

After the early events described above, the process of forming the final shape of the adult leg continues to progress through the subsequent pupal stage, which lasts another 3.5 days or more, but much of its mechanism remains to be elucidated. Although several aspects of the shape-making process, such as changes in the external shape and formation of joints and pulvilli, have been previously reported (Mirth and Akam 2002; Mirth 2005; Tajiri et al. 2010, 2011; Kimura et al. 2020), it is still unknown how individual cells continuously change their shape to form the final shape of the adult tarsus. In this paper, our results of long-term live imaging, in which tarsal morphogenesis during the pupal stage is continuously observed, are described. We found unexpected and dramatic shape changes in epithelial cells and the basement membrane, as well as interesting behavior of macrophage-like cells. The results presented here will greatly contribute to elucidating the mechanism of the final shape formation of the adult tarsus.

## Materials and methods

### Fly strains

Flies were raised on a standard cornmeal/agar/yeast medium at 25 °C unless stated otherwise. The fly strains used were the following: *His2Av-mRFP*^*III.1*^ and *His2Av-mRFP*^*II.1*^ (Pandey et al. 2005), *Jupiter-GFP*^*G00147*^ (Morin et al. 2001), *sGMCA*^*3.1*^ (Kiehart et al. 2000), *sqh-GFP*^*C-42*^ (Royou et al. 2004), *Ubi-GFP-CAAX*^*ZH-22A*^ (Kondo and Hyashi 2013), *Ubi-TagRFP-T-CAAX, LanB1-GFP*^*fTRG00681.sfGFP-TVPTBF*^ (Sarov et al. 2016), UAS*-Apoliner* (Bardet et al. 2008), UAS*-GFP.S65T*, UAS-*GFPnls*, UAS*-TagBFP*^*9D*^, UAS*-Kaede* (Ando et al. 2002), *Ay-*GAL4 (Ito et al. 1997), *Dll-*GAL4^em212^ (Gorfinkiel et al. 1997), *He-*GAL4 (Zettervall et al. 2004), *neur*-GAL4^A101^ (Jhaveri et al. 2000), *srp*Hemo*-*GAL4 (Brückner et al. 2004), and *hsp70-FLP*. Further details on fly strains are found in Flybase (http://flybase.org). Genotypes of flies used for each figure and movie are shown in Table S1.

### Live imaging and image analysis

For live imaging after 12 h APF (i.e., Stage III onwards), the puparium was completely removed. The pupa covered only by the pupal cuticle was mounted with the ventral side down on the bottom of a glass-bottom dish. This allowed us to observe the tarsi of the first and second legs from their lateral sides since they are oriented laterally so that their lateral sides face toward the outside of the pupa. We essentially observed the tarsus of the first leg. A small amount of water or silicon oil (Olympus) was put between the glass-bottom and the pupa. Silicon grease (Shin-Etsu Silicone) was placed around the pupa, on which a cover glass was placed. In this preparation, we succeeded in keeping flies alive until the end of the pupal stage. Z stack images were acquired at regular time intervals.

In Stage II (before 12 h APF), the puparium cannot be removed without affecting development due to the incomplete formation of the pupal cuticle or its tight association with the puparium. In addition, the tissue movement is very rapid. Therefore, the pupa without its puparium removed was set up as described above for live imaging, and we collected images of a single *XY* plane corresponding to the longitudinal section every 2 min.

All live imaging observations were performed using the inverted confocal microscope, FV3000 (Olympus) at room temperature (not strict but near 25 °C). Images were analyzed by ImageJ (https://fiji.sc/), Imaris (Oxford Instruments), and cellSens (Olympus).

### Mosaic analysis

White pupae of flies carrying *hsp70-FLP*, *Ay-*GAL4, and UAS*-GFP.S65T* were collected into a new culture vial and heat shocked by soaking the culture vial in a water bath incubator for 30 min at 37 °C. After heat shock, the flies were allowed to develop at 25 °C. Z stack images were obtained between 18 h APF and 19.5 h APF as described above. The number of protrusions in each cell was counted manually.

### UV-photoconversion of Kaede

Pupae carrying *Dll-*GAL4^em212^ and UAS*-Kaede* were mounted as described above at 17 h APF. Prior to live imaging, the 405-nm laser was irradiated to cells at nearly constant intervals for 5 s. In all irradiations, green signals of Kaede were immediately photoconverted to red signals. Then, live imaging was started from 18 h APF as described above.

### Observation of hemocyte markers

For observation of *He*>*GFPnls*, pupal legs of flies carrying *He-*GAL4 and UAS*-GFPnls* were dissected in phosphate-buffered saline (PBS). Then, it was fixed in PBS containing 4% Paraformaldehyde for 1.5 h in room temperature and washed in PBS containing 0.01% Saponin (PBSS). After that, they were stained in PBSS containing 0.25% 4′,6-Diamidine-2′-phenylindole dihydrochloride (SIGMA) and 0.5~0.75% Alexa Fluor 647 Phalloidin (Invitrogen) for 3 h. Stained samples were washed in PBSS and mounted with VECTASHIELD (Vector Labs). Images were obtained by FV1000 (Olympus). Observations of s*rp*Hemo>*GFP* flies were carried out in the same way as other live imaging experiments.

## Results

### Overview of the final shape formation of the Drosophila tarsus during the pupal stage

**Fig. 1:**
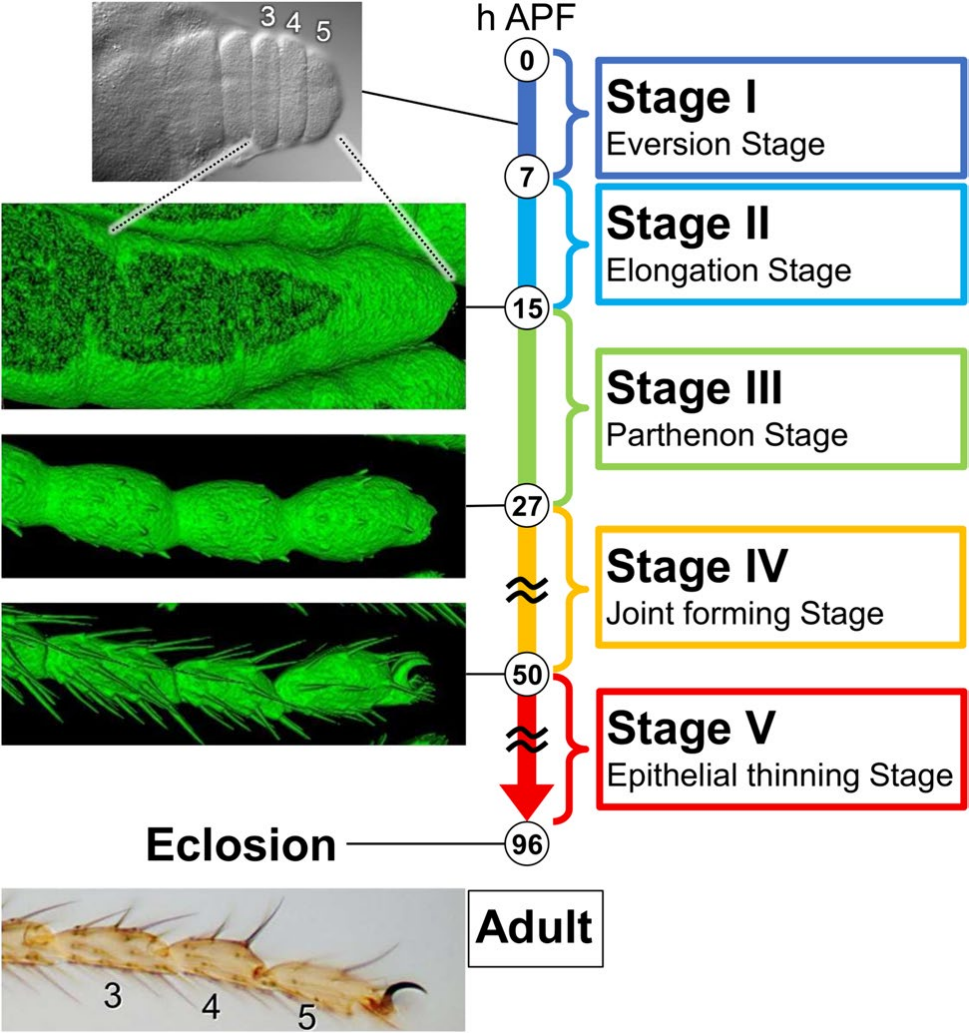
The overview of the final shape formation of *Drosophila* adult tarsus during the pupal stage. We divided the entire process into five stages based on the morphological changes at both tissue and cellular levels observed in long-term live imaging experiments. Representative photos or surface-rendered images (sGMCA signal) corresponding to several time points are shown on the left side. See also Supplemental Movies S1, S3

To observe tarsal development during the pupal stage in detail, we took advantage of long-term live imaging. How the final shape of the tarsus is formed through very dynamic changes at both tissue and cellular levels was successfully traced. Together with earlier observations, we divide the entire process into the following five stages for convenience (Fig. 1).

#### Stage I (0–7 h APF)—eversion stage

Just after puparium formation (APF), the leg disc partially elongates and evaginates from the center, making each tarsal segment recognizable morphologically. This process has been reported to be controlled by cell shape changes, cell rearrangements, and the interaction between the disc epithelium and peripodial epithelium (Condic et al. 1991; Proag et al. 2019). As this process has been described in detail previously, we do not get into it in this paper.

#### Stage II (7–15 h APF)—elongation stage

After the eversion of the leg disc, the elongation of the tarsus further proceeds by unfolding of the morphologically distinctive tarsal segments. By 15 h APF, the tarsus fully elongates, with the tarsal segments no longer visible morphologically, forming a bloated tube with a large lumen diameter. Both the length and diameter of the tarsus reach their maxima. The latter is more than three times the diameter of the adult tarsus (Fig. S1B). The pupal cuticle is formed at least by the end of this stage.

#### Stage III (15–27 h APF)—Parthenon stage

The tarsus decreases its diameter dramatically (Fig. S1B). During this change, indentations between tarsal segments reappear and deepen, making segmental structures visible again. Moreover, some bristles start to sprout by the end of this stage. The name of this stage comes from an interesting structure of the epithelial cells seen in this stage, which we named the Parthenon-like structure. Its details are described later.

#### Stage IV (27–50 h APF)—joint forming stage

The indentation between tarsal segments deepens further, and the joint formation between tarsal segments proceeds. Bristles fully elongate, and their formation is nearly completed. Furthermore, the formation of pretarsal structures, such as claws and pulvilli occurs mainly in this stage. By about 50 h APF, the formation of the external shapes of the adult leg is almost complete.

#### Stage V (50–96 h APF)—epithelial thinning stage

Seen from the outside, the shape of the leg hardly changes during this stage. However, we discovered that the thickness of the epithelial cell layer continues to reduce gradually. Thus, we separated this stage from Stage IV as an independent stage.

Hereafter, we describe the details of cell dynamics in each of the above stages except Stage I.

### Stage II—elongation stage

In Stage II, the tarsus elongates to its maximum length along the proximodistal direction to form a broad and straight tubular structure. Live imaging was performed without removing the puparium in Stage II since it is difficult to remove the puparium without causing developmental defects before 12 h APF. In this condition, the depth of the focus at which clear images could be obtained was limited. In addition to this limitation, the tissue elongates so quickly that taking images as a Z series was also difficult. Accordingly, we acquired images of a single *XY* plane corresponding to the longitudinal section every 2 min. We used flies expressing GFP-CAAX (labeling the plasma membrane) and His2Av-mRFP (labeling the nuclei). At the beginning of the live imaging, 7 h APF, the everted tarsus was slightly elongated but the folding of each tarsal segment was still visible (Fig. S2A, Supplemental Movie S2). From around 9 h APF, the elongation of the tarsus was accelerated with the rapid unfolding of each tarsal segment. By 11 h APF, the folding disappeared almost completely, and the tarsus had become a bloated, straight epithelial tube (Fig. S2B–E). During this process, epithelial cells initially long in the apicobasal direction became cuboidal in shape.

Interestingly, a cluster of large cells was observed coming from the proximal side and spreading distally in the lumen of the elongating tarsus (Fig. S2B–E, Supplemental Movie S2). After this stage onwards, they were always observed in the tarsus (see below). Since these large cells expressed hemocyte-specific GAL4, such as *He-*GAL4 or *srp*Hemo*-*GAL4, they appeared to be hemocytes (Fig. S3), presumably macrophage-like cells according to their morphology and behavior (see below).

### Stage III—Parthenon stage

From this stage onwards, the pupa becomes resilient to having its puparium removed and can develop into the adult after the complete removal of the puparium. The developing pupa without the puparium is only covered by the transparent pupal cuticle, which allows us to acquire clear images at relatively deeper focuses. Additionally, from Stage III onwards, changes in overall shape are no longer as rapid as in Stage II. Accordingly, we could collect Z series images at each time point during long-term live imaging experiments from Stage III (Supplemental Movie S1, S3). This long-term live imaging experiment allowed us to analyze detailed cellular behaviors. As mentioned above, the overall diameter of the tarsus decreases dramatically in Stage III. We observed that surprisingly dynamic changes in cell morphology occur inside during this process. Around 18 h APF, epithelial cells deformed their shapes and temporarily formed an interesting structure. We named it the Parthenon-like structure because it reminded us of the Parthenon, an ancient Greek ruin. Details of the Parthenon-like structure and associated cellular dynamics are described in the following three subsections.

#### The architecture of the Parthenon-like structure

**Fig. 2:**
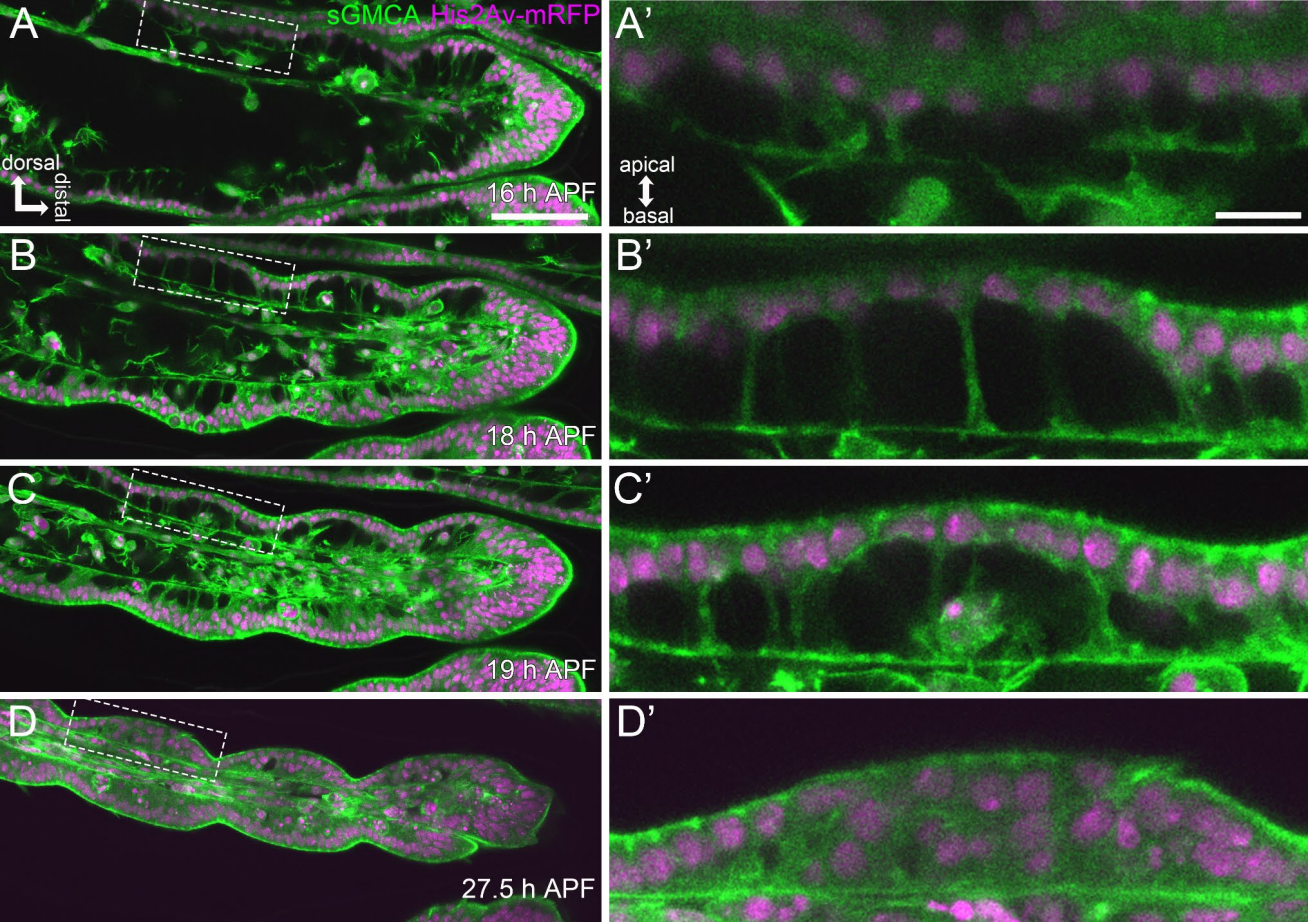
Dynamic shape changes in the tarsal epithelial cells in Stage III. **A, B, C, D** Stills from the live imaging of the distal part of the tarsus in the sGMCA (green) and His2Av-mRFP (magenta) expressing fly. The tissue rapidly narrows, and the indentation of joint regions progresses. **A’**, **B’**, **C’**, **D’** Magnification of regions surrounded by the dashed lines in **A, B, C,** **D**, respectively. Apicobasal projections are seen in **A’**, **B’**, **C’** and become difficult to distinguish in **D’**. Distal is to the right and dorsal to the top in all figures. Stages are shown at the lower right corners in **A, B, C, D**. Scale bars in **A** and **A’**, 50 μm for **A, B, C, D** and 10 μm for **A’**, **B’**, **C’**, **D’**, respectively. See also Supplemental Movie S3

**Fig. 3:**
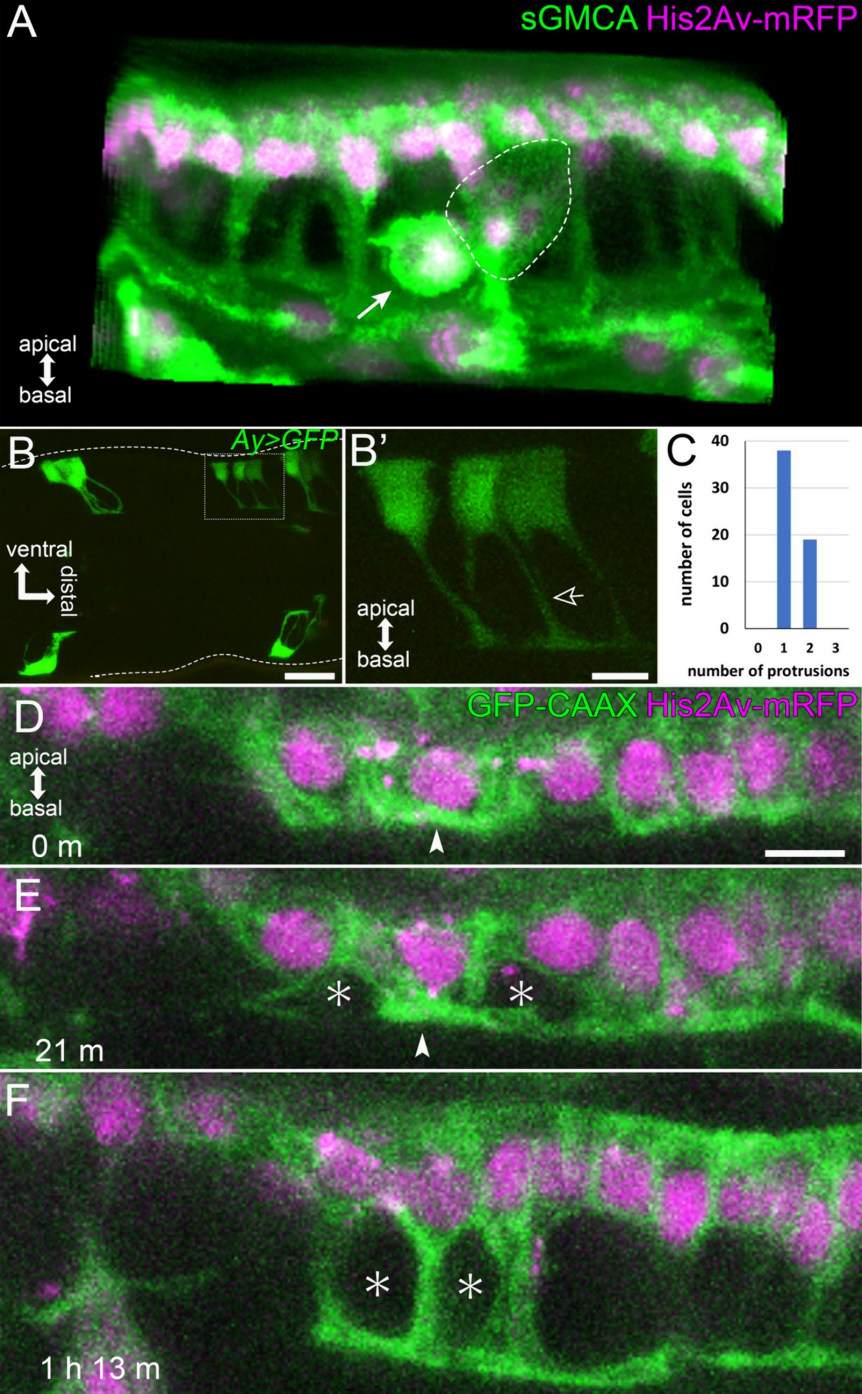
Architecture of the Parthenon-like structure. **A** The reconstructed image of epithelial cells forming the Parthenon-like structure in the sGMCA (green) and His2Av-mRFP (magenta) expressing fly. Nuclei and most volumes of sGMCA signals are aligned as a monolayer on the apical (outer) side. Pillar-like sGMCA signals are elongated in the apicobasal direction. At the basal (inner) side, sGMCA signals spread planarly, creating a thin layer. Macrophage-like cells are found in the cavities between the pillars (arrow). Several clusters of cells whose cell bodies and nuclei fall into cavities are seen (dashed line). These cells were suggested to be sensory organ cells (see also Fig. S4). **B**, **B’** GFP-expressing cell clusters obtained in the mosaic analysis. **B** Mosaic GFP expression in the distal part of the tarsal segment 2 and proximal part of the tarsal segment 3 in 19 h APF induced in the *Ay*>*GFP* fly. Dashed lines indicate the outline of the leg. **B’** Reconstructed image of the four GFP-expressing epithelial cells corresponding to cells surrounded by the dotted rectangle in **B**. The leftmost cell has one protrusion, while the one next has two. One of the protrusions extending from the second cell from the left appears to form a bundle with a protrusion extending from a neighboring cell (open arrow). See also Supplemental Movie S4. **C** Number of protrusions in each GFP-expressing cell. About two-thirds of epithelial cells had one protrusion, and one-third of them had two. **D**–**F** Stills from the live imaging of tarsal epithelial cells initiating the Parthenon-like structure formation. Cell membrane is labelled by GFP-CAAX (green), and nuclei are labelled by His2Av-mRFP (magenta). Triangular-shaped cavities are observed at the initiation of the process (asterisks in **E**, **F**). Strong signals of GFP-CAAX are observed where the pillars are formed (arrowheads in **D**, **E**). The elapsed time from **D** are indicated at the lower left corners. See also Supplemental Movie S5. Apical is to the top in **A**, **B’**, and **D**–**F**. Scale bars in **B** and **B’**, 20 μm and 5 μm, respectively. Scale bar in **D**, 5 μm for **D**–**F**

Firstly, flies expressing His2Av-mRFP (labeling the nuclei) and sGMCA (labeling actin) were observed, and Z series images were obtained every 15 min (Fig. 2, Supplemental Movies S1, S3). By reconstructing a 3D structure using a part of these images obtained at 18 h APF, we found a very curious architecture of the epithelial cell layer (Fig. 3A). Nuclei and most volumes of sGMCA signals were aligned as a monolayer on the apical (outer) side. At the basal (inner) side, sGMCA signals spread planarly, creating a thin layer. Between these two layers, pillar-like sGMCA signals were seen connecting them. There appeared to be open spaces or cavities between the pillars. Since sGMCA labels actin, it could be possible that epithelial cells are tightly lined up without space between them as usual and that it is the cytoskeleton forming the curious structure. However, similar structures were also observed in flies expressing plain GFP (labeling cytosol) under the control of *Distal-less* (*Dll*)-GAL4 (*Dll>GFP*) (Fig. S4A) and GFP-CAAX (labeling cell membrane) (see Fig. 3D–F).Thus, we concluded that epithelial cells themselves form this remarkable structure by deformation; that is, epithelial cells tightly align most of their cell body containing nuclei at the apical side, protrude cytoplasm as a pillar-like process in a basal direction, and then spread at the basal side, making cavities within the epithelial cell layer. As mentioned above, we named this structure the Parthenon-like structure after the ancient Greek ruin. We also observed the Parthenon-like structure in flies expressing Jupiter-GFP (labeling microtubule) and Sqh-GFP (labeling Myo II) (Fig. S4B, C). Thus, cytoskeletal molecules, such as actin, microtubule, and Myo II, appear to be assembled in cables inside the pillars. Within the Parthenon-like structure, several clusters of cells whose cell bodies and nuclei fell into cavities were observed (surrounded by a dashed line in Fig. 3A). The time-lapse observations until later stages and labeling of sensory cells by TagBFP driven by *neuralized* (*neur*)-GAL4, which is specifically expressed in the sensory cells, indicate that these cells are sensory organ cells (Fig. S4D–D”). Interestingly, we found that macrophage-like cells frequently existed and were actively moving around in the cavities between pillars (arrow in Fig. 3A). Their detailed behavior is described later.

To reveal whether the pillar-like structures are contributed by all the epidermal cells or only a part of them, we carried out the mosaic analysis by the flip-out technique using flies having *hsp70-FLP*, UAS-*GFP*, and *Ay*-GAL4 (Golic and Lindquist 1989; Ito et al. 1997), in which small patches of epithelial cells were labeled by GFP (Fig. 3B). The reconstructed image of the representative cluster of four epithelial cells is shown in Fig. 3B’ and Supplemental Movie S4. The leftmost cell had one protrusion while the next one on the right had two. Moreover, the right protrusion of the second cell looked to be making a bundle with a protrusion from the adjacent third cell on the right of the second cell (open arrow in Fig. 3B’, Supplemental Movie S4). When counting how many protrusions each cell extended, we found that, in 18 h APF, all epithelial cells extended one or two protrusions (Fig. 3C; only one protrusion in about two-thirds of the epithelial cells and two in about one-third). These results indicate that all epithelial cells contribute to the pillars of the Parthenon-like structure and that each pillar is made up of protrusions derived from multiple adjacent cells.

#### Formation and disappearance of the Parthenon-like structure

As shown above, the shape of epithelial cells was cuboidal at the end of Stage II (Fig. S2E, Supplemental Movie S2). These cuboidal epithelial cells started to form rapidly the Parthenon-like structure with the onset of Stage III (Fig. 2A’, B’). The thickness of the epithelial cell layer rapidly increased with the elongation of the pillars of the Parthenon-like structure (Fig. S1C). Once the epithelial thickness reached its maximum, the Parthenon-like structure turned to be resolved rapidly by the shortening of the pillars and disappearance of the cavities (Fig. 2C’, D’), resulting in the decrease of the epithelial thickness (Fig. S1C). During the above process, the lumen diameter and overall diameter of the tarsus also reduced dramatically (Fig. S1B), implying a close relationship between the formation/disappearance of the Parthenon-like structure and the reduction of the tarsus diameter. The disappearance of the Parthenon-like structure progressed faster in the future joint region than in the inter-joint region, resulting in the invagination of the apical surface (see arrowheads in Supplemental Movie S3). This allowed the morphological distinction of tarsal segments again.

To reveal details in the Parthenon-like structure formation, Z stack images were obtained every 5 min using flies expressing GFP-CAAX and His2Av-mRFP (Fig. 3D–F, Supplemental Movie S5). Before the formation of the Parthenon-like structure initiated, cuboidal epithelial cells were aligned as a monolayer (Fig. 3D). In around 16 h APF, at the very beginning of the formation of the Parthenon-like structure, a part of the cytoplasm of epithelial cells protruded basally, while connections between neighboring cells at the basal surface looked to be maintained, resulting in the formation of triangular-shaped cavities (Fig. 3E, F, asterisks). In addition, relatively strong signals of GFP-CAAX were observed in the area where the pillars were being formed (Fig. 3D. E arrowheads). The protrusions then elongated basally to form the pillars (Fig. 3F).

#### The basal surface of the Parthenon-like structure

**Fig. 4:**
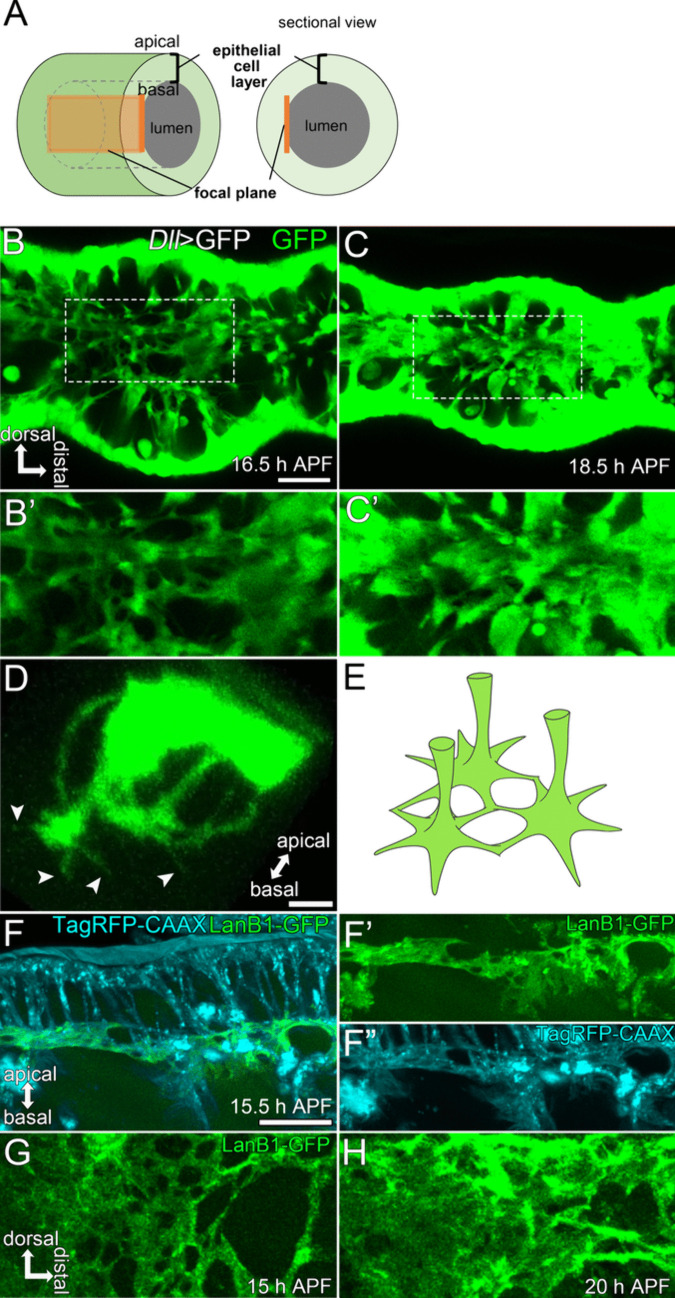
Basal-meshwork shrinkage of the Parthenon-like structure. **A** Schematic drawing showing the position of the focal plane to observe the basal surface of epithelial cells in **B**–**C’**, **G**, **H**. **B**–**C’** Stills from live imaging of the basal surface of the tarsal segment 3 using the *Dll*>*GFP* fly. Regions surrounded by the dashed lines in **B** and **C** are magnified in **B’** and **C’**, respectively. Many holes can be seen in **B’**, suggesting the basal surface forms a mesh-like structure. The holes become less visible in **C’**. See also Supplemental Movie S6. **D** Reconstructed image of a GFP-expressing cluster of tarsal epithelial cells viewing down on the basal side from an oblique angle. Thin projections are seen at the basal surface (arrowheads). See also Supplemental Movie S7. **E** Schematic drawing of the basal meshwork hypothesized from **B’**, **C’**, and **D**. **F**–**F”** Simultaneous observation of TagRFP-CAAX (cyan) and LanB1-GFP (green) at the basal meshwork. The positions of holes are largely shared between LanB1-GFP (**F’**) and TagRFP-CAAX (**F”**) signals. **G**, **H** Stills from live imaging of the basal surface of the tarsal segment 3 in the LanB1-GFP expressing fly, which are corresponding to **B’**, **C’**. See also Supplemental Movie S8. Stages are indicated at the lower right corners in **B**, **C**, **F, G, H**. Distal is to the right and dorsal to the top in **B**–**C’**, **G**, **H**. Scale bars in **B**, 20 μm for **B**, **C**, 10 μm for **B’**, **C’**, **G**, **H**, in **D**, 5 μm, in **F**, 10 μm for **F**–**F”**

As the lumen diameter dramatically reduced during the formation and disappearance of the Parthenon-like structure, we thought that some structural changes in the basal part of the Parthenon-like structure must occur during this process. Thus, we focused on the structure and behavior of the basal surface of epithelial cells. To know its detailed structure, we observed the focal planes including only the basal surface of GFP-expressing epithelial cells (the floor of the Parthenon-like structure) in *Dll>GFP* flies (Fig. 4A–C’). Surprisingly, in 16.5 h APF, the basal surface of epithelial cells showed a mesh-like pattern and had many holes (Fig. 4B’). The live imaging showed these holes rapidly shrank and almost disappeared within 2 h, as the overall diameter of the tarsus reduced (Fig 4B’, C’, Supplemental Movie S6). To reveal how each epithelial cell comprises the basal meshwork, we carefully analyzed clusters of epithelial cells expressing GFP in *Ay*>*GFP* flies. Observation of 3D-reconstructed images of clusters showed that filopodia-like thin protrusions were elongated from the roots of the pillars (Fig. 4D, Supplemental Movie S7). According to this observation, we hypothesized that such filopodia-like protrusions connect to each other to form the basal meshwork as shown in the illustration in Fig. 4E.

Generally, with the basal surface of epithelial cells, the basement membrane is in contact. To investigate the structure of the basement membrane during the shrinkage of the basal meshwork of epithelial cells, we examined the basement membrane structure using flies expressing Laminin B1-GFP (LanB1-GFP) and RFP-CAAX (Fig. 4F–H, Supplemental Movie S8). LanB1 is a major component of the basement membrane. LanB1-GFP signals coincided with the basal surface of epithelial cells, as shown by the comparison between LanB1-GFP and RFP-CAAX signals (Fig. 4F–F”). The basement membrane also showed the mesh-like structure at the initiation of the Parthenon-like structure formation (Fig. 4G). The meshwork of LanB1-GFP signals also shrank, becoming uniform and membranous signals, as is generally seen (Fig. 4H). These observations indicate that the basement membrane structure also changed dramatically during the formation and disappearance of the Parthenon-like structure.

**Fig. 5:**
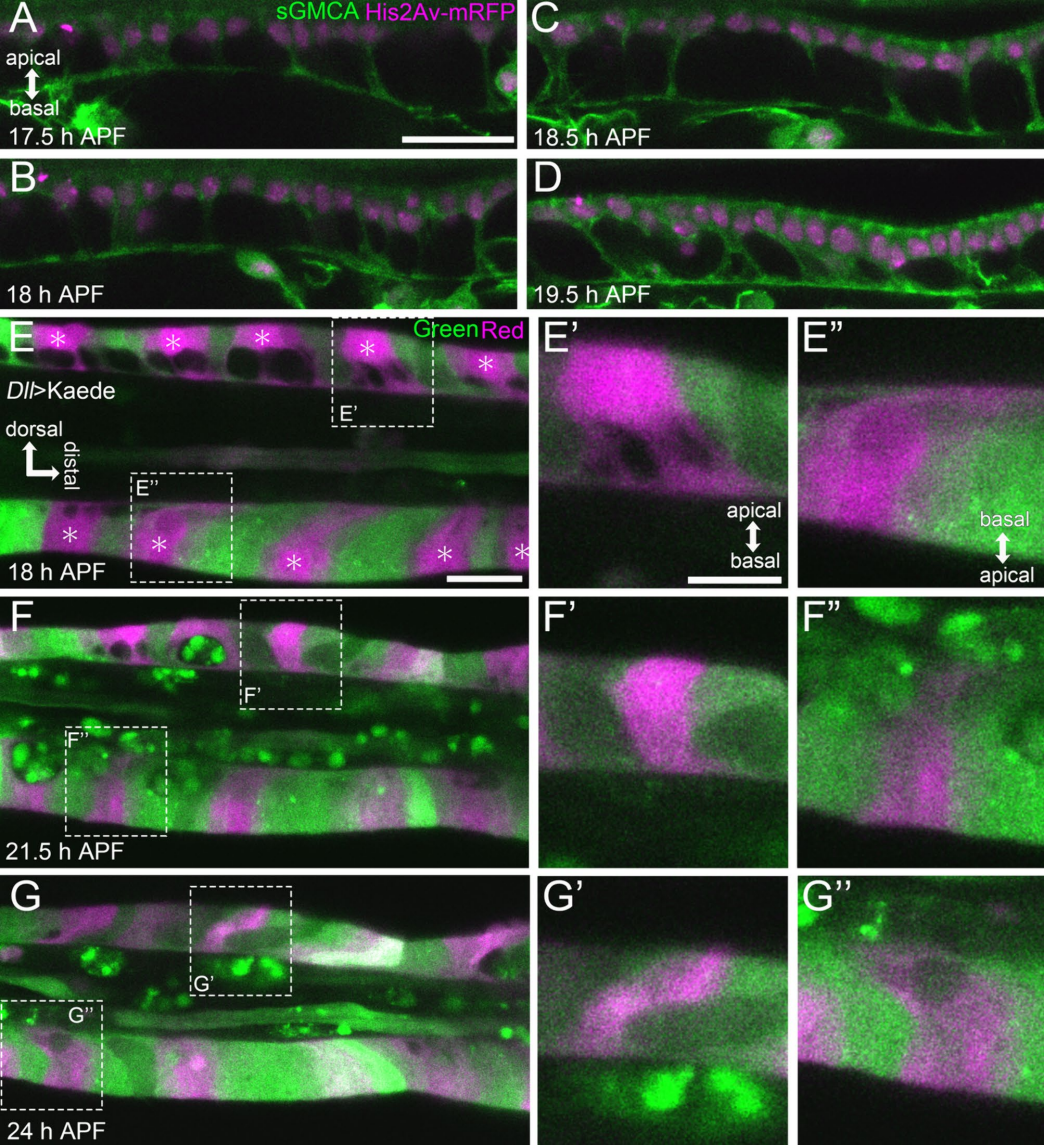
Increased tension at the basal surface of epithelial cells along the proximodistal axis in Stage III. **A**–**D** Stills from the live imaging of tarsal epithelium of the sGMCA (green) and His2Av-mRFP (magenta) expressing fly. The winding basal surface in **A** progressively flattened in **B**–**D**. **E**–**G”** Stills from the live imaging of a *Dll*>*Kaede* fly. Foci of UV irradiation are indicated by asterisks in **E**. Selected photoconverted regions (dashed rectangles in **E**, **F**, **G**) in the dorsal and ventral epithelium are magnified in **E’**, **E”**, **F’**, **F”**, **G’**, **G”**, respectively. Initially, the basal side of epithelial cells are located distally than the apical side (**E**–**E”**), then pulled proximally (**F**–**F”**), and finally, the relative position of the apical and basal sides inverted (**G**–**G”**). See also Supplemental Movie S9. Stages are indicated at the lower left corners in **A**–**E**, **F**, **G**. Distal is to the right and dorsal to the top in all figures. Note that in **E”**, **F”**, **G”**, apical is to the bottom since they are ventral epithelium. Scale bars in **A**, 20 μm for **A**–**D**, in **E**, 20 μm for **E, F, G**, in **E’**, 5 μm for **E’, E”, F’, F”, G’, G”**

In addition to the meshwork to membranous change in the basal surface of the Parthenon-like structure, we found another interesting behavior of it. As shown in Fig. 5A, the basal surface of epithelial cells was winding along the proximodistal axis immediately after the Parthenon-like structure started to form, possibly because the progression of the pillar elongation varies from place to place along the proximodistal axis. However, it progressively flattened along with the shrinkage of the basal meshwork (Fig. 5B, C). In 19.5 h APF, the basal surface of epithelial cells was very straight and taut (Fig. 5D). These observations imply increasing tension on the basal surface in the proximodistal direction. We further obtained interesting data related to the proximodistal tension by utilizing the photoconvertible fluorescent protein Kaede, which converts the color from green to red in response to UV irradiation (Ando et al. 2002). We irradiated epithelial cells positioned at roughly regular intervals with UV light and then, observed the tarsus by live imaging (Fig. 5E–G”, Supplemental Movie S9). Since the tarsus is a three-dimensional structure, and epithelial cells are arranged three-dimensionally, it was difficult to irradiate only one target cell. Instead, several neighboring cells were labeled simultaneously in each of the UV-irradiated loci. In 18 h APF, pillars of the Parthenon-like structure appeared to be tilted relative to the apicobasal axis, with the basal side more distal than the apical side (Fig. 5E–E”). Interestingly, live imaging showed that the basal side then moved significantly toward the proximal direction (Fig. 5F–F”, Supplemental Movie S9). By 24 h APF, the tilting direction of the pillars was reversed, so that the basal side was more proximal than the apical side (Fig. 5G–G”). These observations also imply that there is a tension that pulls the basal part of the Parthenon-like structure proximally.

### Stage IV—joint forming stage

**Fig. 6:**
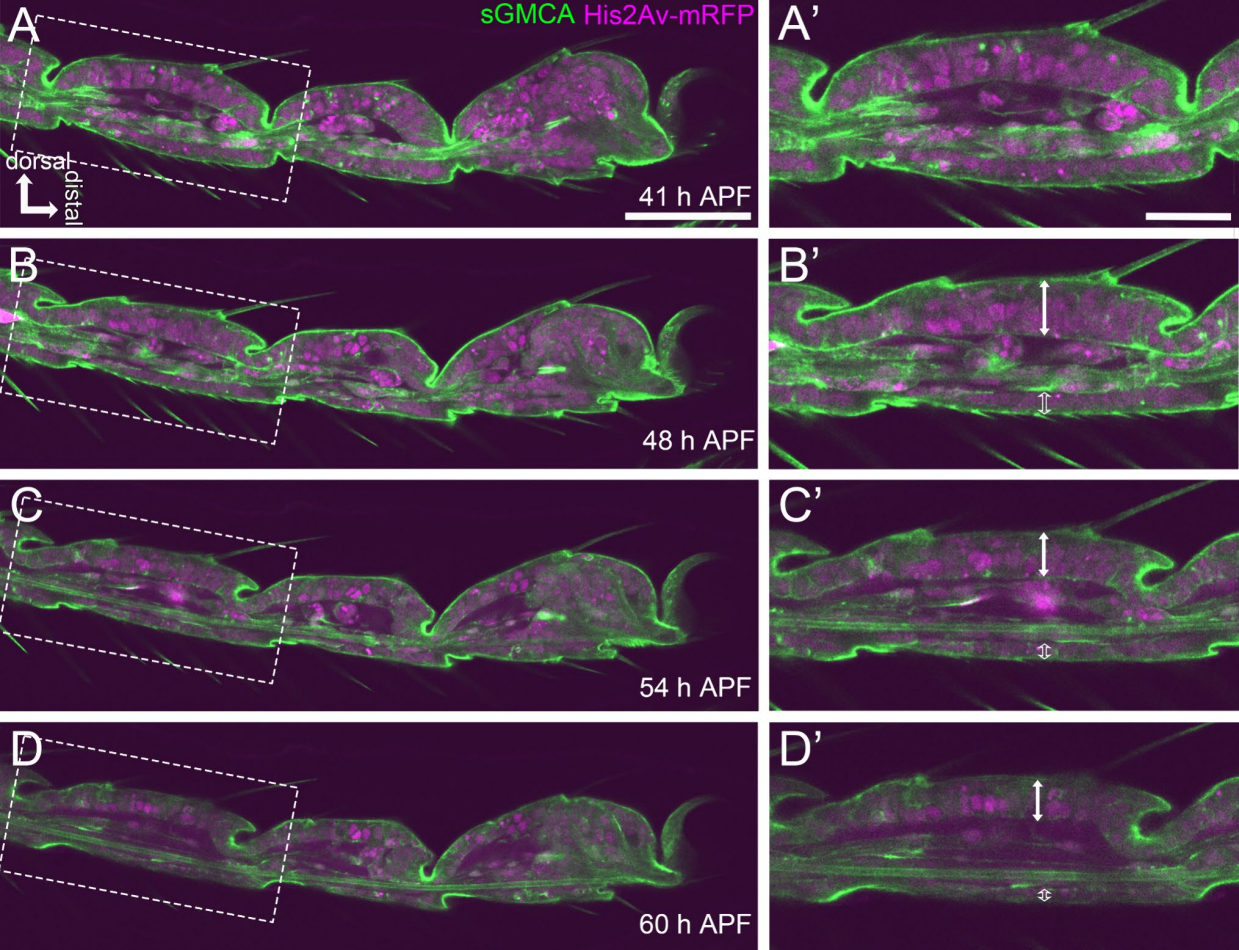
Shape changes in the tarsal epithelial cell layer in Stages IV and V. **A, B, C, D** Stills from the live imaging of the distal part of the tarsus in the sGMCA (green) and His2Av-mRFP (magenta) expressing fly. **A**, **B** are in Stage IV, and **C**, **D** are in Stage V. Dashed rectangles in **A, B, C, D** are magnified in **A’**, **B’**, **C’**, **D’**, respectively. By the end of Stage IV (**A**–**B’**), the external shape formation, such as elongation of bristles, claws, and pulvilli are almost complete. The bottom of the invaginated joint moved proximally, and then, the ball-and-socket structure formation progressed in Stage V. The morphological difference between the dorsal and ventral epithelium became apparent (double arrows and open double arrows in **B’**, **C’**, **D’**). In Stage V (**C**–**D’**), the epithelial layer decreased its thickness without hardly changing the outline of segments. See also Supplemental Movie S10. Dorsal is to the top and distal to the right in all figures. Scale bars in **A**, 50 μm for **A, B, C, D**, in **A’**, 20 μm for **A’**, **B’**, **C’**, **D’**

By the end of this stage, the shaping process of the external morphology of the adult leg, such as the elongation of bristles and formation of claws and pulvilli, was almost complete (Fig. 6A, B, Supplemental Movie S1, S3). The details of the pulvilli formation have been described previously by Kimura et al. (2020). From this stage onwards, the difference between the dorsal and ventral sides of the tarsus became apparent. The thickness of the epithelial cell layer became much thinner on the ventral side than on the dorsal side (Fig. S1C). The ventral epithelial cell layer was straight, whereas the dorsal epithelial cell layer was somewhat arched (Fig. 6A–B’). Furthermore, the formation of the ball-and-socket structure of the joint was started on the dorsal side but not on the ventral side (Fig. 6B–C’). On the dorsal side, invagination of the joint region became deepened with a greater reduction of the luminal diameter at the joint region than in the inter-joint region, making a deep cleft between neighboring tarsal segments (Fig. 6A, A’, Supplemental Movie S3). After the cleft reached its deepest at around 40 h APF, the bottom of the cleft moved proximally (Fig. 6B, B’, Supplemental Movie S3, S10), and then, the ball-and-socket structure formation of the joint proceeded during the early Stage V (Fig. 6C’, D’, Supplemental Movie S3, S10). This process has previously been described in detail by Tajiri et al. (2010). The arched dorsal epithelial cell layer seemed to result from the deep invagination at the joint region. In Stage IV, the overall diameter of each segment was slightly reduced but not so much as in Stage III (Fig. S1B).

### Stage V—epithelial thinning stage

Even after the completion of the external shape by the end of Stage IV, changes in the epithelial cell layer continued inside. The epithelial cell layer decreased its thickness without hardly changing the outline of segments. During this process, the basal surface of the epithelial cell layer was getting closer to the apical surface (Fig. 6C–D’, Supplemental Movie S10).

### The behavior of macrophage-like cells

**Fig. 7:**
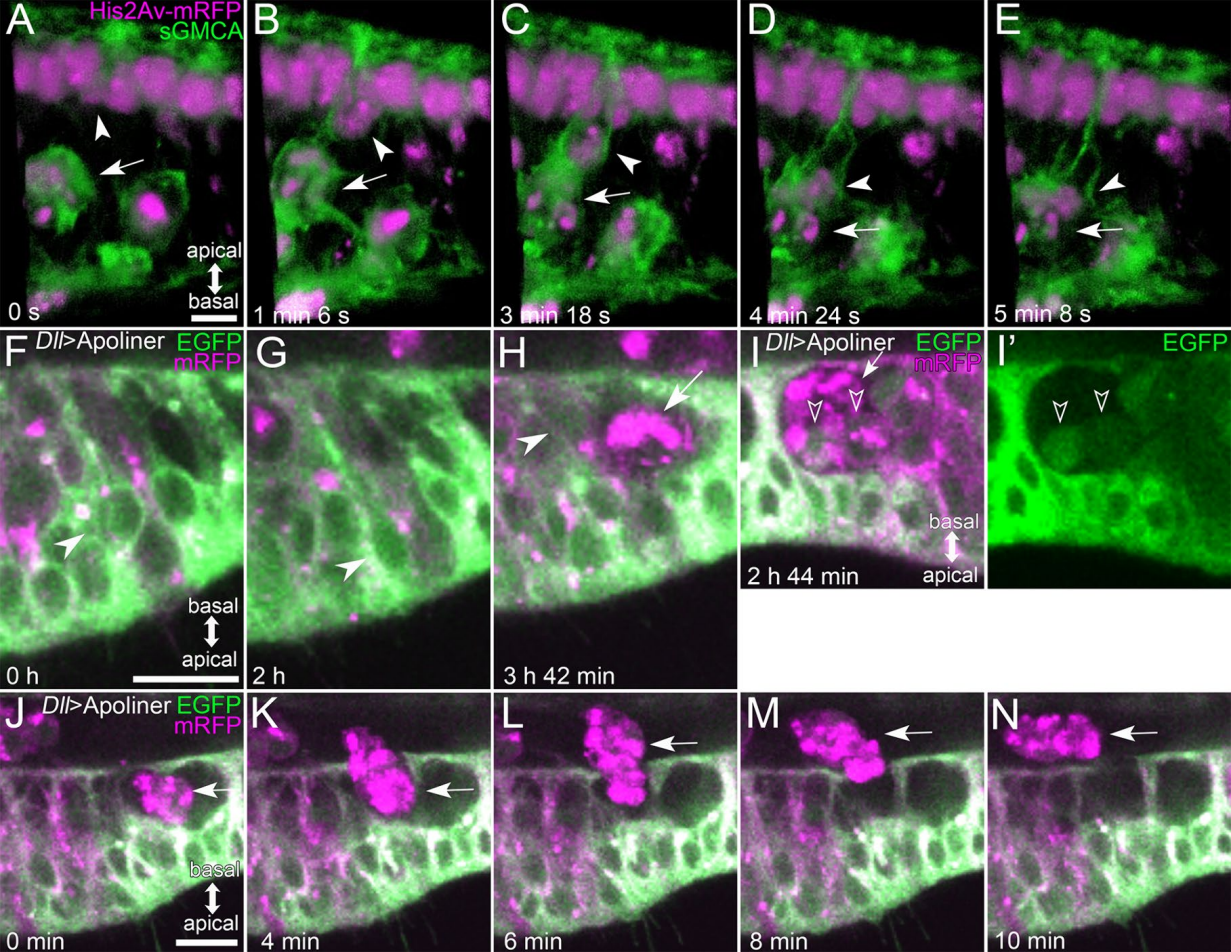
Behavior of macrophage-like cells in Stage III. **A**–**E** Stills from the reconstructed movie of the live imaging of the tarsal epithelium in Stage III in the sGMCA (green) and His2Av-mRFP (magenta) expressing fly. The macrophage-like cell located in the cavity of the Parthenon-like structure (arrows) engulfs the epithelial cell being extruded from the apical layer (arrowheads). **F**–**N** Stills from the live imaging of ventral tarsal epithelium in Stage III using *Dll*>*Apoliner* flies. In cells with caspase activity, mRFP signals are retained at the plasma membrane but EGFP signals move to nuclei. Macrophage-like cells are seen as clusters of large dots of mRFP signals (arrows in **H** and **I**). **F**–**H** The epithelial cell with caspase activity OFF (arrowhead in **F**) eventually turns the caspase activity ON (arrowhead in **G**), and then moves to the basal side (arrowhead in **H**), where a macrophage-like cell appears (arrow in **H**). See also Supplemental Movie S12. **I**, **I’** A macrophage-like cell (arrow in **I**) appearing to have engulfed epithelial cells with caspase activity ON (open arrowheads in **I**, **I’**). **I’** EGFP channel extracted from **I**. **J**–**N** A macrophage-like cell located in the cavity of the Parthenon-like structure (arrows) popes out to the lumen through a presumptive hole of the basal meshwork (see also Supplemental Movie S13, Fig. 4). Apical is to the top in **A**–**E**, to the bottom in **F**–**N**. Scale bars in **A**, 5 μm for **A**–**E**, in **F**, 10 μm for **F**–**I’**, in **J**, 10 μm for **J**–**N**

As shown above, we observed that macrophage-like cells were getting into the lumen of the expanding tarsus from the proximal end in Stage II (see Fig. S2, Supplemental Movie S2) and resided in the cavity of the Parthenon-like structure, where they were actively moving around, in Stage III (see Fig. 3 and Supplemental Movie S3). Fascinatingly, by obtaining Z stack images every 22 s, we succeeded in capturing the moment when a macrophage-like cell in the Parthenon-like structure phagocytoses an epithelial cell (Fig. 7A–E, Supplemental Movie S11). A macrophage-like cell (arrow in Fig. 7A–E) in the cavity of the Parthenon-like structure extended filopodia apically and phagocytosed an epithelial cell whose cell body containing the nucleus was extruded basally (arrowheads in Fig. 7A–E). To confirm that cells phagocytosed by macrophage-like cells were apoptotic cells, we observed the caspase activity in epithelial cells by expressing Apoliner under the control of *Dll*-GAL4 (*Dll*>*Apoliner*). Apoliner is a caspase activity reporter, in which mRFP with a transmembrane domain is connected to EGFP with nuclear localization signal (NLS-EGFP) by a linker containing the target site cleaved by caspases. Without caspase activity, both mRFP and EGFP are colocalized on the cell membrane, while NLS-EGFP is released from the membrane-attached mRFP and gets into the nucleus in response to caspase activity (Bardet et al. 2008). Since we used *Dll*-GAL4 to drive Apoliner expression, it was expressed only in epithelial cells but not in macrophage-like cells. However, we could see a macrophage-like cell as a cluster of relatively large dots of mRFP signals (Fig. 7H–N) probably due to the accumulation of mRFP derived from engulfed epithelial cells, as mentioned in the original report (Bardet et al. 2008). This helped us to find macrophage-like cells in *Dll*>*Apoliner* flies. Through live imaging, we observed that one epithelial cell, which initially located its cell body at the apical side without the nuclear EGFP signal, began to show the EGFP signal in its nucleus (Fig. 7F, G), and then, its cell body migrated in a basal direction (Fig. 7H), finally reaching a macrophage-like cell detected as a cluster of mRFP signals (Fig. 7H, see also Supplemental Movie S12). Macrophage-like cells with multiple EGFP signals were often observed (Fig. 7I, I’). These signals appeared to reflect EGFP signals in nuclei of engulfed epithelial cells. In addition, we also successively observed a macrophage-like cell passing through the basal surface of the Parthenon-like structure (Fig. 7J–N, Supplemental Movie S13). Taken together, it appears that macrophage-like cells move in and out of the cavities of the Parthenon-like structure, and when entering the cavity, they phagocytose epithelial cells initiating apoptosis.

## Discussion

Taking advantage of long-term live imaging, we could reveal nearly the whole shaping process of the adult tarsus during the pupal stage in *Drosophila*. Changes in the shape of cells and the tissue as a composite of cells were more dramatic and complex than previously thought. Epithelial cells change their shape from columnar to cuboidal during the elongation of the tarsus in Stage II (see Fig. S2 and Supplemental Movie S2). Then, the cuboidal epithelial cells further change their shape dramatically to form the unexpected structure, the Parthenon-like structure, in Stage III (see Fig. 3 and Supplemental Movie S5). The Parthenon-like structure is a transient structure, and the rapid reduction in the tarsal diameter occurs during the formation and disappearance of the Parthenon-like structure (see Fig. S1B and Supplemental Movie S3). After the tarsal diameter becomes that of the adult leg at the end of Stage III, the thickness of the epithelial cell layer continues to reduce in Stage IV and Stage V (see Fig. S1C, Fig. 6 and Supplemental Movie S10). Especially in Stage V, although the external shape is completed, the thinning of the epithelial cell layer further continues internally. In addition, we could also document the behavior of macrophage-like cells (see Fig. 7 and Supplemental Movie S11-13). The possible mechanisms and roles of these cellular dynamics are discussed below.

### Mechanism of the tarsal elongation in Stage II

In Stage II, the everted tarsus fully elongates to its maximum length. The invasion of macrophage-like cells from proximal to distal during the tarsal elongation (see Fig. S2, Supplemental Movie S2) makes us imagine the influx of hemolymph into the tarsus. It may be possible that the influx of hemolymph contributes to the tarsal elongation by raising inner pressure. Previously, it has been proposed that the tarsal elongation is a result of hydrostatic pressure, according to the fact that the tarsal elongation can be caused by pressing on the abdomen of the prepupa (Fristrom and Fristrom 1993). Therefore, hydrostatic pressure may be one of the primary forces driving the tarsal elongation in Stage II. Interestingly, it has been shown that the elongation of the horn during the pupal stage in the beetle is caused by hydrostatic pressure (Matsuda et al. 2017). Thus, this may be one of the fundamental mechanisms for the rapid elongation of long, rod-like appendages.

Interestingly, it has been reported that the degradation of several basement membrane proteins in Stage I is important for the elongation and cell shape changes (Diaz-de-la-Loza et al. 2018). Therefore, the combination of the release of epithelial cells from the constraining force provided by the basement membrane and the increase in hydrostatic pressure may be important.

### Mechanism of the transient formation of the Parthenon-like structure

Epithelial cells comprising the Parthenon-like structure apically align the most of their cell body containing the nuclei, elongate the cytoplasmic process in a basal direction, and connect with each other at the basal end to form a thin layer, making the cavity in the epithelial cell layer (see Fig. 3). Each epithelial cell protrudes one or two processes, and the processes of neighboring cells bundle together to form the pillar. This is reminiscent of neurons extending axons and forming axon bundles. Thus, it might be possible that just as neurons extend axons and then connect with other cells, epithelial cells protrude processes first and then form the basal connection. When observing the initial step of the Parthenon-like structure formation, however, we found that the basal connection between epithelial cells already formed in the very beginning (see Fig. 3D–F). This may indicate that epithelial cells are basally connected before initiating the Parthenon-like structure formation and protrude processes while maintaining the connection, unlike neurons.

Since actin, microtubule, and Myo II were abundantly found in the pillars of the Parthenon-like structure (see Fig. S4), cytoskeletal regulation may actively contribute to the formation and disappearance of the Parthenon-like structure by controlling elongation and retraction of the pillars. As for the formation of the Parthenon-like structure, another but not exclusive idea is the contribution of the basement membrane. Evidence that the basement membrane actively contributes to tissue shape has been accumulating recently (reviewed in Morrissey and Sherwood 2015; Pastor-Pareja 2020; Töpfer 2023). In the case of our observation on the tarsal development, both the basal surface of epithelial cells and the basement membrane initially showed a mesh-like pattern, and they shrank to form the membranous structure without holes during the reduction of the luminal diameter (see Fig. 4). This may indicate that changes in the composition of the basement membrane produce a force to pull epithelial cells toward the basal side and contribute to the elongation of the pillars, i.e., the formation of the Parthenon-like structure.

### Disappearance and reappearance of the epithelial folds prefiguring the adult leg joints

Before Stage II, the tarsal joints of the adult leg are prefigured by the folding of the epithelium. Interestingly, our observation showed that the epithelial folding is resolved by the end of Stage II and reappears during Stage III along with the disappearance of the Parthenon-like structure. This raises the question of how the identity of the joint regions is maintained. Notch signaling and its target genes such as *AP-2* and *dysfusion* are required for both the early folding and the later refolding. Accordingly, the expression of Notch ligands or its target genes in the joint regions is detected prior to the early folding as well as prior to the later refolding (de Celis et al. 1998; Kerber et al. 2001; Córdoba and Estella 2014; Tajiri et al. 2011). These insights are consistent with a model in which continuous Notch signaling in the joint regions, maintained by sustained expression of the ligands, regulates both the early folding and the later refolding. Meanwhile, considering the extensive cell dynamics during the long interval between the two phases of folding, the possibility that Notch activity is remodeled or refined during the interval cannot be excluded. The present study will be the basis for unraveling how cell identities are maintained in the face of dynamic cell behaviors.

### Function of macrophage-like cells

In Stage II, macrophage-like cells were observed entering the lumen of the elongating tarsus from the proximal side and spreading distally (see Fig. S2, Supplemental Movie S2). This observation may indicate that macrophage-like cells that originate outside the leg come with the hemolymph flow. After entering the tarsal lumen, macrophage-like cells continued to reside and were actively moving around in the lumen (see Supplemental Movie S3). When the Parthenon-like structure is formed, they intruded into and moved around in the cavity (see Fig. 7, Supplemental Movie S13). Within the cavity, they phagocytosed epithelial cells with caspase activity (see Fig. 7A–I’). According to these observations, macrophage-like cells might be involved in controlling cell number by phagocytosing excess cells. Making the cavity within the epithelial cell layer by forming the Parthenon-like structure might help macrophage-like cells engulf epithelial cells.

Previously, it has been reported that the depletion of hemocytes by specifically inducing cell death results in severe morphological defects, such as truncation in legs (Arefin et al. 2015). Macrophage-like cells may also play another role in tarsal morphogenesis besides the trimming of excess cells.

### Generality of the Parthenon-like structure

The Parthenon-like structure does not seem to be a special structure observed only in the tarsus. We found it also in other leg segments (Fig. S5A). Moreover, a similar structure could be observed in the head region and the notum region (Fig. S5B-E). In the wing, epithelial cells in the pupal stage are known to form similar shapes with epithelial cells in the Parthenon-like structure, in which cell bodies are aligned apically and protrude the thin process basally, although the structure of the basal side seems to be different from the Parthenon-like structure (Waddington 1940; Fristrom et al. 1993; Sun et al. 2021; Tran et al. 2024). Furthermore, in the pupal wing of lepidopteran insects, epithelial cells with similar protrusion and hemocytes moving around between them have been reported (Nardi and Magee-Adams 1986; Kodama et al. 1995; Ohno and Otaki 2015; McDougal et al. 2021). Other than the wing, structures resembling the Parthenon-like structure have also been found in the prepupal abdominal epidermis and developing pupal antennae of Lepidoptera (Delhanty and Locke 1989; Ando et al. 2011). Therefore, the transient formation of the Parthenon-like structure appears to be one of the essential processes of final shape formation by epithelial cells at least in insects. Further study on the mechanism of the transient formation of the Parthenon-like structure and its role in morphogenesis will help to better understand the mechanisms of final shape formation.

## Supplementary information

ESM 1 (PDF 1379 kb)

ESM 2 **Supplemental Movie S1** Surface-rendered movie of the long-term live imaging of the distal part of the tarsus throughout Stage III-V. The sGMCA signals of the same dataset as Supplemental Movie S3 were reconstructed. Dramatic shape changes of the whole tissue are shown. Time stamp shows hh:mm. Distal is to the right and dorsal to the top. See also Fig. 1, Supplemental Movie S3. **Supplemental Movie S2** Live imaging of the tarsal elongation and macrophage invasion into the lumen in Stage II corresponding to Fig. S2. The distal part of the tarsus was observed from 7 h APF. The cell membrane and nuclei are labeled by GFP-CAAX (green) and His2Av-mRFP (magenta), respectively. Segmental foldings are visible at 7 h APF, however, they progressively become invisible as the leg elongates. A cluster of large moving cells, presumably macrophage-like cells, are seen entering the lumen from the proximal part. Distal is to the right and ventral is to the top. **Supplemental Movie S3** Long-term live imaging of the distal part of tarsus throughout Stage III-V in the sGMCA (green) and His2Av-mRFP (magenta) expressing fly. The most representative XY plane was extracted from the same dataset as Supplemental Movie S1. Arrowheads indicate examples of future joint regions. Time stamp shows hh:mm. Distal is to the right and dorsal to the top. See also Fig. 1, 2, Supplemental Movie S1. **Supplemental Movie S4** Reconstructed movie of the four GFP-expressing epithelial cells obtained in the mosaic analysis in the *Ay*>*GFP* fly corresponding to Fig. 3B’. The 3D structure of the cells is shown in rotation. Apical is to the top. **Supplemental Movie S5** Live imaging of tarsal epithelial cells initiating the Parthenon-like structure formation corresponding to Fig. 3D-F. Cell membrane and nuclei are labelled by GFP-CAAX (green) and His2Av-mRFP (magenta), respectively. Time stamp shows hh:mm:ss. Apical is to the top. **Supplemental Movie S6** Live imaging of the basal surface of the tarsal segment 3 in the *Dll*>*GFP* fly, corresponding to Fig. 4B’, C’. The focal planes, including only the basal surface of epithelial cells, were selected from each time point. Holes seen in the early time points are progressively shrunk. Time stamp shows hh:mm. Dorsal is to the top and distal to the right. **Supplemental Movie S7** A reconstructed movie of a GFP-expressing epithelial cell cluster was obtained in the mosaic analysis using the *Ay*>*GFP* fly corresponding to Fig. 4D. The 3D structure of the cells is shown in rotation. Thin filopodia are seen at the basal side. Apical is diagonally upward to the right. **Supplemental Movie S8** Live imaging of the basal surface of the tarsal segment 3 in the LanB1-GFP expressing fly corresponding to Fig. 4G, H. The focal planes, including only the basal surface of epithelial cells, were selected from each time point. Holes seen in the early time points are progressively shrunk. Time stamp shows hh:mm. Dorsal is to the top and distal to the right. **Supplemental Movie S9** Live imaging of the photoconvertible protein Kaede expressing tarsus corresponding to Fig. 5E-G”. The regions shown in magenta are photoconverted by UV irradiation. Many protrusions are elongated distally (right) from the apical cell bodies at 18 h APF, however, they shift the orientation proximally. Dorsal is to the top and distal to the right. **Supplemental Movie S10** The region corresponding to Fig.6A’, B’, C’, D’ was extracted from Supplemental Movie S3. In the late Stage IV (40-50 h APF), the bottom of the cleft formed by joint invagination moves proximally, and then, the ball-and-socket structure formation progresses. In the early Stage V (50-60 h APF), the epithelial cell layer decreases its thickness, hardly changing the outline of segments. Time stamp shows hh:mm. Dorsal is to the top and distal to the right. **Supplemental Movie S11** A reconstructed movie of the sGMCA and His2Av-mRFP expressing tarsal epithelium corresponding to Fig. 7A-E. Images were obtained every 22 sec from 16.5 h APF. The macrophage-like cell in the space between pillars of the Parthenon-like structure extends filopodia to engulf the epithelial cell being extruded basally. Time stamp shows mm:ss. Apical is to the top. **Supplemental Movie S12** Live imaging of ventral tarsal epithelium in Stage III in the *Dll*>*Apoliner* fly corresponding to Fig. 7F-H. Images were obtained every 2 min from 19 h APF. At first, the cell marked by an arrowhead is located at the apical side without the nuclear EGFP signal. After about two hours, it begins to show EGFP signal and then moves to the basal side. Finally, it seems to be engulfed by a macrophage-like cell, which is visualized by the accumulated mRFP signals. Time stamp shows hh:mm. Basal is to the top and distal to the right. **Supplemental Movie S13** live imaging of ventral tarsal epithelium in Stage III in the *Dll*>*Apoliner* fly corresponding to Fig. 7J-N. Images of a different region at 21.5 h APF were extracted from the same data as Supplemental Movie S12. A macrophage-like cell (arrowhead) at the cavity of the Parthenon-like structure, which is visualized by the accumulated mRFP signals, passes through a hole on the basal surface of the epithelium to enter the lumen. Basal to the top and distal to the right. (ZIP 119697 kb)

## Data Availability

No datasets were generated or analyzed during the current study.
