# Supplementary material for: Unveiling the cell dynamics during the final shape formation of the tarsus in Drosophila adult leg by live imaging
Source: Dev Genes Evol. 2024 Jul 8;234(2):117–33. doi: 10.1007/s00427-024-00719-z (PMC11611951; doi:10.1007/s00427-024-00719-z)
Supplement: Supplementary file 1 — (PDF 1379 kb) [file 427_2024_719_MOESM1_ESM.pdf]

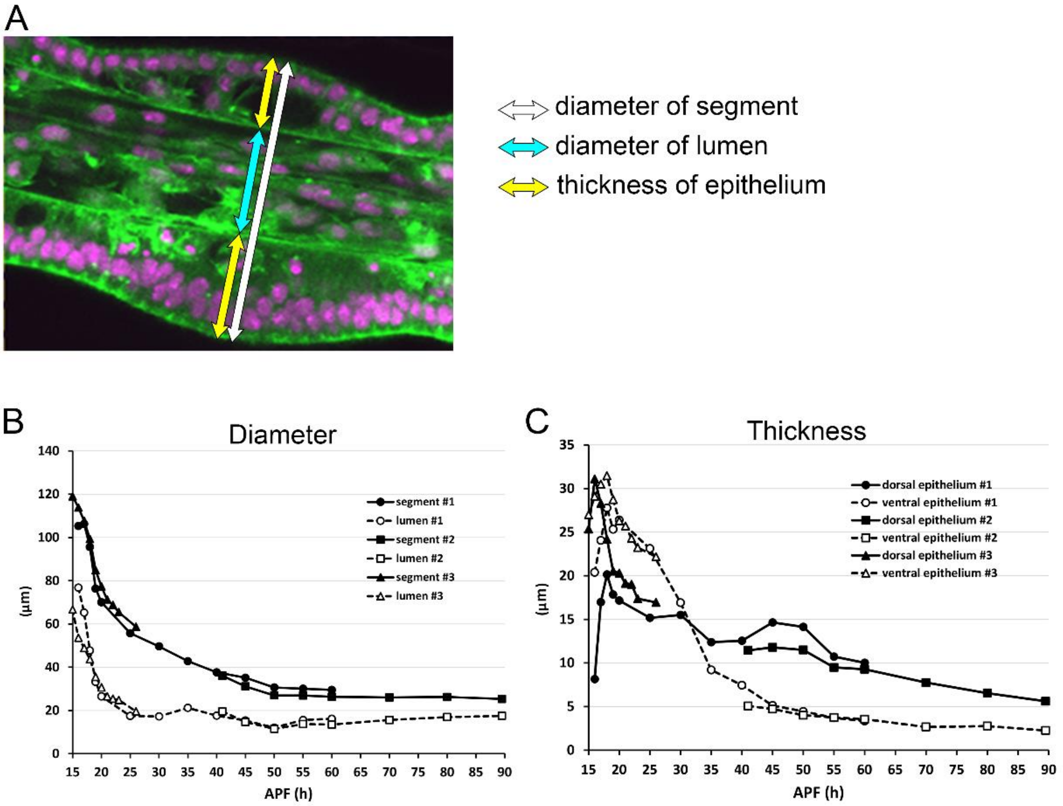

Fig. S1 Measurements of diameter and epithelial thickness of the tarsal segment 3. (**A**) Schematic explanation of the measurement showed in (**B**, **C**). The picture shows the dorsoventral section of the tarsal segment 3 in the pupal stage. The section in the middle of the tissue (i.e. showed maximum segmental diameter) was selected to be measured. The dorsoventral diameter of the segment (white double arrow) and thickness of the epithelium (yellow double arrow) were manually measured using ImageJ. The diameter of the lumen (blue double arrow) was calculated as the subtraction of the dorsal and ventral epithelial thickness from the segmental diameter. (**B**) Dorsoventral diameter of the whole tissue (solid lines) and lumen (dashed lines) measured from live imaging data. (**C**) The thickness of the dorsal (solid lines) and the ventral (dashed lines) epithelial cell layers measured from live imaging data. The plots connected by lines are sequential live imaging data. The genotypes used in each live imaging are shown in **Tabel S1**

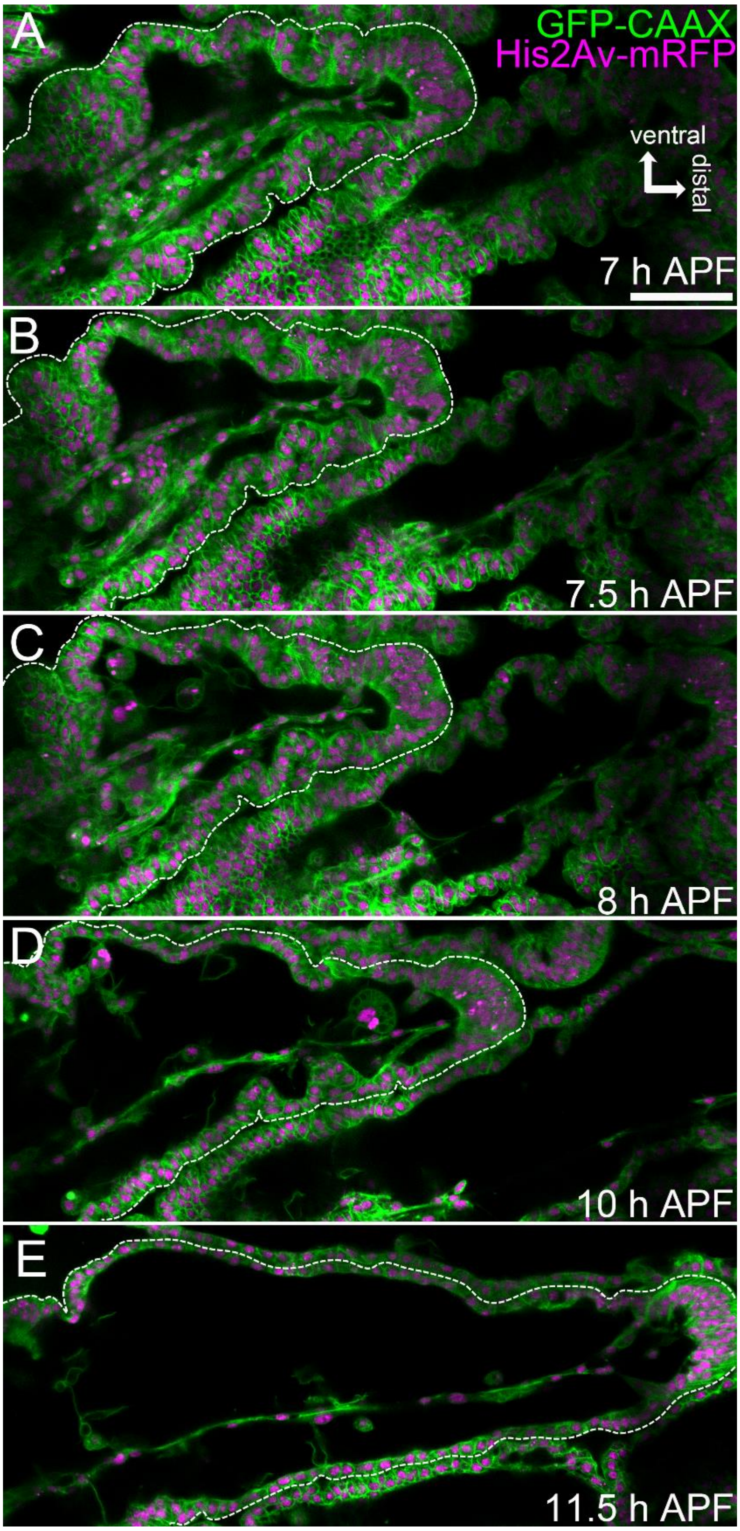

Fig. S2 Tarsal elongation and macrophage invasion into the lumen in Stage II. (**A-E**) Stills from live imaging of the distal part of the tarsus in the GFP-CAAX (green) and His2Av-mRFP (magenta) expressing fly. The dashed line outlines the tarsus of one leg. Segmental foldings are visible at 7 h APF (**A**), however, they become almost invisible as the tissue progressively elongated (**B-E**). A cluster of large cells, presumably macrophage-like cells, are seen entering the lumen from the proximal part of the tarsus. See also **Supplemental Movie S2**. Ventral is to the top and distal to the right in all figures. Scale bar in (**A**), 50 µm for all figures

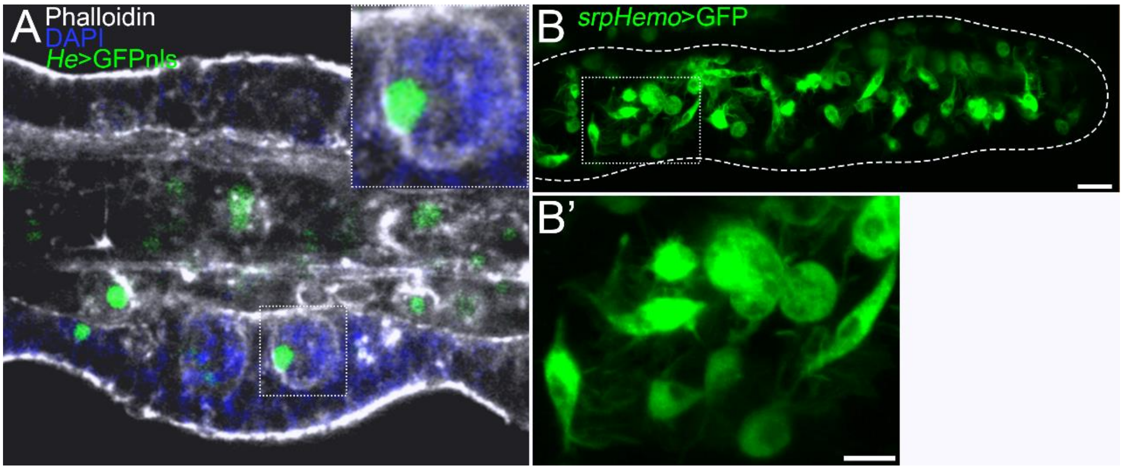

Fig. S3 Expression of hemocyte markers (**A**) The tarsal segment 3 of the *He>GFPnls* fly at 19 h APF stained with Phalloidin (white) and DAPI (blue). The region surrounded by a dotted line is magnified in the inset. The spherical cell, shown in the inset, had one GFP positive nucleus and multiple GFP negative nuclei, suggesting that it is a macrophage-like cell engulfed several epithelial cells. (**B**) A still from the live imaging of the distal part of the tarsus in the *srp.Hemo>GFP* fly at 15 h APF. All Z slices were projected. Dashed line shows the outline of the tarsus. Many GFP expressing cells are observed inside the tarsus. The region surrounded by the dotted line is magnified in (**B’**). (**B’**) Most GFP-expressing cells extended filopodia, consistent with the presumption that they are macrophage-like cells. Scale bars, 20 µm in (**B**) and 10 µm in (**B’**)

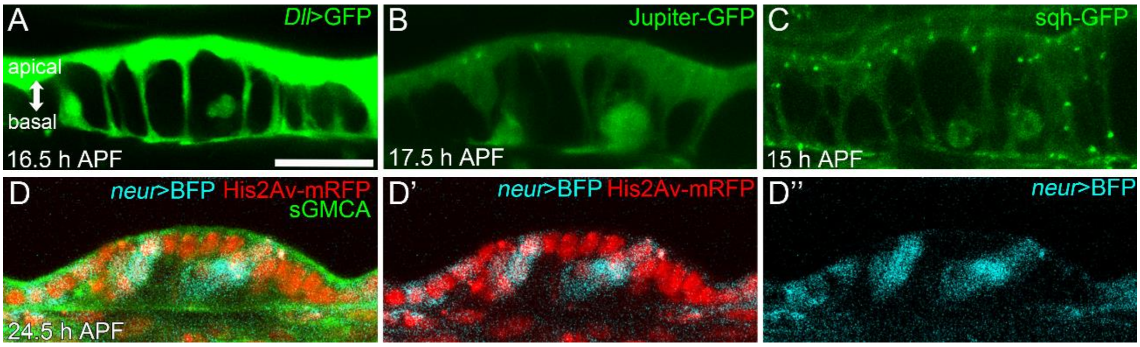

Fig. S4 Observation of the Parthenon-like structure by several reporters. (**A-C**) Observation of the Parthenon-like structure in *Dll>GFP* (**A**), *Jupiter-GFP* (**B**), *sqh-GFP* (**C**) flies. The Parthenon-like structure appears to be formed by shape-change of epithelial cells. (**D-D"**) Simultaneous observation of *neur*>BFP, His2Av-mRFP, sGMCA in the Parthenon-like structure. BFP signals are observed in cells whose nuclei are clustered inside the cavities of the Parthenon-like structure, suggesting that these are sensory organ cells. Scale bar in (**A**), 20 μm for all figures. Apical is to the top in all figures

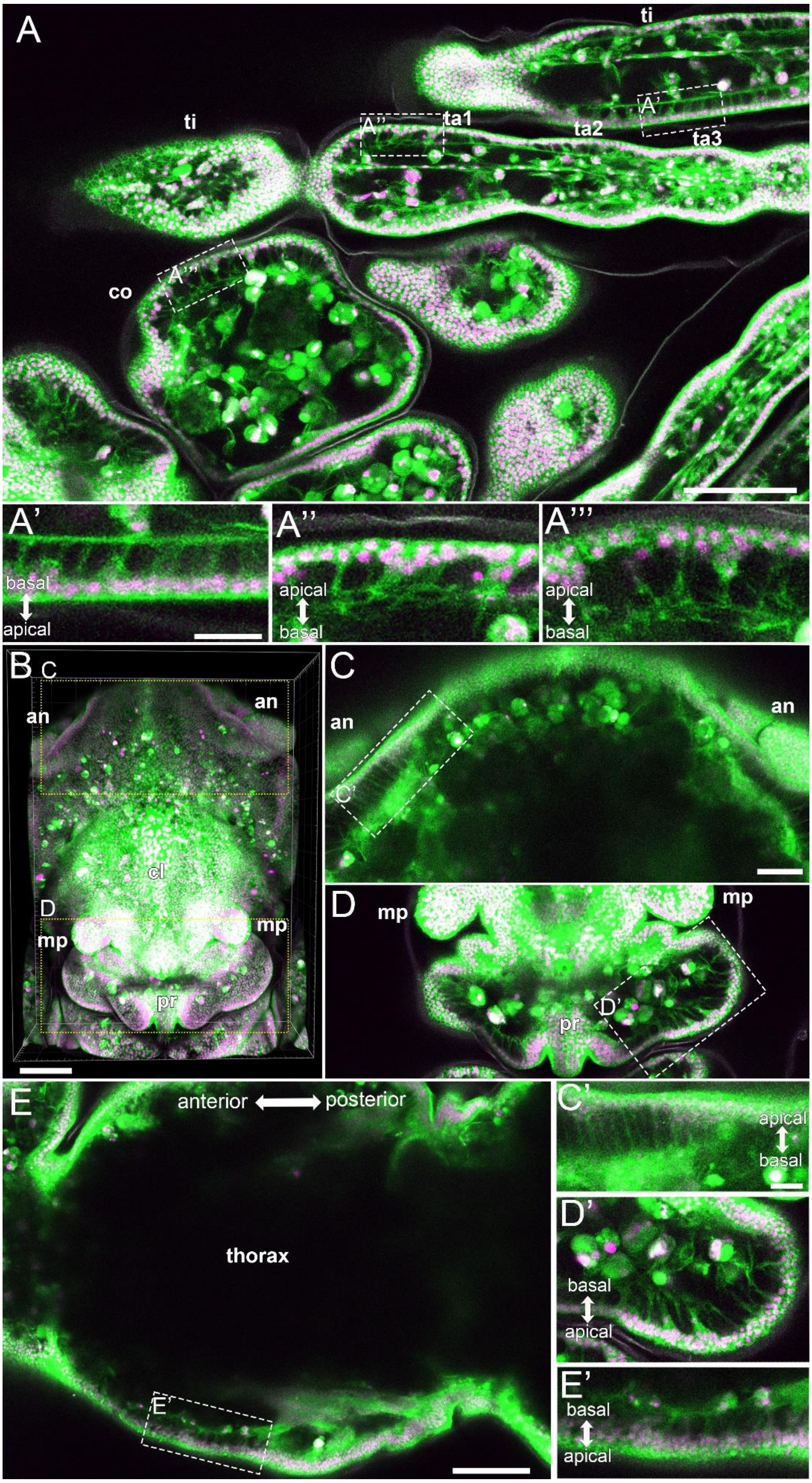
A
ti
ti
ta1
ta2
ta3
co
A'
A''
A'''
A'
basal
apical
A''
apical
basal
A'''
apical
basal
B
C
an
an
cl
D
mp
mp
pr
C
an
an
C'
D
mp
mp
pr
D'
E
anterior
posterior
thorax
E'
C'
apical
basal
D'
basal
apical
E'
basal
apical

Fig. S5 Observation of the Parthenon-like structure in various tissues other than the tarsus. (**A**) the ventral view of the proximal leg segments of the sGMCA (green) and His2Av-mRFP (magenta) expressing fly at 20 h APF. Dashed rectangles are enlarged in (**A'-A'''**). The Parthenon-like structure was seen in not only the proximal part of the tarsus (**A'**) but also in other leg segments such as the tibia (**A''**) and coxa (**A'''**). ta: tarsal segment, ti: tibia, co: coxa. (**B**) the frontal view of volume-rendered head region of the sGMCA (green) and His2Av-mRFP (magenta) expressing fly at 23 h APF. The sections corresponding to dotted rectangles are enlarged in (**C**, **D**). The dashed rectangle regions in (**C**, **D**) are enlarged in (**C'**, **D'**), respectively. The structures very similar to the Parthenon-like structure are seen in the epithelium near the antenna (**C'**) and of the proboscis (**D'**). an: antenna, cl: clypeus, mp: maxillary pulps, pr: proboscis. (**E**) the dorsal view of the thorax of the sGMCA (green) and His2Av-mRFP (magenta) expressing fly at 23.5 h APF. The dashed rectangle is enlarged in (**E'**). The thoracic epithelium also shows basally elongated protrusions like the Parthenon-like structure. Scale bars in (**A**), 100 μm, in (**A'**), 20 μm for (**A'-A'''**), in (**B**), 100 μm, in (**C**), 50 μm for (**C**, **D**), in (**E**), 100 μm, in (**C'**), 20 μm for (**C'-E'**). Apical is to the top in (**A''**, **A'''**, **C'**) and to the bottom in (**A'**, **D'**, **E'**)

Table S1. Detailed genotypes of flies in each figure

Figure	Genotype
Fig. 2	*w*^{1118}; *His2Av-mRFP*$^{III, 1}$, *sGMCA*$^{3, 1}$
Fig. 3A	*w*^{1118}; *His2Av-mRFP*$^{III, 1}$, *sGMCA*$^{3, 1}$
Fig. 3B, B’	*hsFLP*^{1}, *w*^{1118}/UAS-*Dcer2*^{1}, *w*^{1118}; *Ay-GAL4*, UAS-*GFPT2*/ *CyO*
Fig. 3D-F	*w**; *Ubi-GFP-CAAX*, *His2Av-mRFP*$^{II.2}$
Fig. 4B, B’, C, C’	*w**; *Dll-GAL4*^{em212}/+, UAS-*GFPT2*/+
Fig. 4D	*hsFLP*^{1}, *w*^{1118}/UAS-*Dcer2*^{1}, *w*^{1118}; *Ay-GAL4*, UAS-*GFPT2*/ *CyO*
Fig. 4F-F”	*y*^{1}, *w**; *Ubi-TagRFP-T-CAAX*/+; *LanB1-GFP*/+
Fig. 4G, H	*LanB1-GFP*
Fig. 5A-D	*w*^{1118}; *His2Av-mRFP*$^{III, 1}$, *sGMCA*$^{3, 1}$
Fig.5E-E”, F-F”, G-G”	UAS-*Dcer2*^{1}, *w*^{1118}; *Dll-GAL4*^{em212}/ UAS-*Kaede. C*^{2}; +/ *TM3*, *Ser*^{1}
Fig. 6	*w*^{1118}; *His2Av-mRFP*$^{III, 1}$, *sGMCA*$^{3, 1}$
Fig. 7A-E	*w*^{1118}; *His2Av-mRFP*$^{III, 1}$, *sGMCA*$^{3, 1}$
Fig. 7F-N, N’	*w**; *Dll-GAL4*^{em212}/ UAS-*Apoliner*^{5}
Fig. S1 #1, 2	*w*^{1118}; *His2Av-mRFP*$^{III, 1}$, *sGMCA*$^{3, 1}$
Fig. S1 #3	*w**; *Dll-GAL4*^{em212}/ UAS-*Apoliner*^{5}
Fig. S2	*y*^{1}, *w**; *Ubi-TagRFP-T-CAAX*
Fig. S3A	*w**; *He-GAL4*^{85}, UAS-*GFPnls*^{8}
Fig. S3B, B’	*y*^{1}, *w**; *srp. Hemo-GAL4*^{3}, UAS-*GFP*^{3}
Fig. S4A	*w**; *Dll-GAL4*^{em212}/+, UAS-*GFPT2*/+
Fig. S4B	*w**; *Jupiter*^{G00147}
Fig. S4C	*y*^{1}, *w**, *cv*^{1}, *sqh*^{AX3}; *sqh-GFP.RLC*^{2}
Fig. S4D-D”	*w**; *His2Av-mRFP*$^{III, 1}$, *sGMCA*$^{3, 1}$/ *neur-GAL4*^{A101}, UAS-*TagBFP*^{9D}
Fig. S5	*w*^{1118}; *His2Av-mRFP*$^{III, 1}$, *sGMCA*$^{3, 1}$

Supplemental Movie S1	w^{1118}; *His2Av-mRFP*$^{III,\ 1}$, *sGMCA*$^{3,\ 1}$
Supplemental Movie S2	w^{*}; *Ubi-GFP-CAAX*, *His2Av-mRFP*$^{II.2}$
Supplemental Movie S3	w^{1118}; *His2Av-mRFP*$^{III,\ 1}$, *sGMCA*$^{3,\ 1}$
Supplemental Movie S4	*hsFLP*^{1}, w^{1118}/UAS-*Dcer2*^{1}, w^{1118}; *Ay-GAL4*, UAS-*GFPT2*/ *CyO*
Supplemental Movie S5	w^{*}; *Ubi-GFP-CAAX*, *His2Av-mRFP*$^{II.2}$
Supplemental Movie S6	*w**; *Dll-GAL4*^{em212}/+, UAS-*GFPT2*/+
Supplemental Movie S7	*hsFLP*^{1}, w^{1118}/UAS-*Dcer2*^{1}, w^{1118}; *Ay-GAL4*, UAS-*GFPT2*/ *CyO*
Supplemental Movie S8	*LanB1-GFP*
Supplemental Movie S9	UAS-*Dcer2*^{1}, w^{1118}; *Dll-GAL4*^{em212}/ UAS-*Kaede. C*^{2}; +/ *TM3*, *Ser*^{1}
Supplemental Movie S10	w^{1118}; *His2Av-mRFP*$^{III,\ 1}$, *sGMCA*$^{3,\ 1}$
Supplemental Movie S11	w^{1118}; *His2Av-mRFP*$^{III,\ 1}$, *sGMCA*$^{3,\ 1}$
Supplemental Movie S12	w^{*}; *Dll-GAL4*^{em212}/ UAS-*Apoliner*^{5}
Supplemental Movie S13	w^{*}; *Dll-GAL4*^{em212}/ UAS-*Apoliner*^{5}
